# Hemoglobin A_2_ Lowered by Iron Deficiency and **α**-Thalassemia: Should Screening Recommendation for **β**-Thalassemia Change?

**DOI:** 10.1155/2013/858294

**Published:** 2013-03-12

**Authors:** Srdjan Denic, Mukesh M. Agarwal, Bayan Al Dabbagh, Awad El Essa, Mohamed Takala, Saad Showqi, Javed Yassin

**Affiliations:** ^1^Department of Medicine, College of Medicine and Health Sciences, United Arab Emirates University, P.O. Box 17666, Al Ain, Abu Dhabi, UAE; ^2^Department of Pathology, College of Medicine and Health Sciences, United Arab Emirates University, P.O. Box 17666, Al Ain, Abu Dhabi, UAE; ^3^Al Ain Hospital, P.O. 1006, Al Ain, Abu Dhabi, UAE

## Abstract

Screening for **β**-thalassemia trait (BTT) relies on measuring hemoglobin (Hb) A_2_. Since multiple factors can affect HbA_2_ levels, the screening can become unreliable. In 1356 healthy Arabs enrolled into a federally funded premarital BTT screening program, the effects of iron deficiency (ID), *α* ^+^-thalassemia trait, gender, smoking, and tribalism on HbA_2_ were studied. The complete blood count and hemoglobin fractions were determined on the entire cohort; serum ferritin (<15 **μ**g/L) in 391 subjects was used to determine ID. BTT was present in 29 (2.1%) subjects (HbA_2_ > 3.5%). Among 77(20.3%) subjects with ID, the mean HbA_2_ (2.30 ± 0.23%) was 0.2% lower than in subjects without iron deficiency (2.50 ± 0.24%, *P* < 0.0001). In 65 (38%)/172 subjects with phenotypic *α* ^+^-thalassemia trait, the mean HbA_2_ (2.43 ± 0.24%) was 0.13% lower than in subjects without *α* ^+^-thalassemia trait, *P* < 0.0001. The mean HbA_2_ did not differ between males and females, smokers and nonsmokers, and between the tribes. Thus, 35 (2.6%) subjects with HbA_2_ between 3.2 and 3.5% were at a risk of false negative diagnosis of BTT. Since iron deficiency and *α* ^+^-thalassemia are both common and both lower HbA_2_, modifications in screening recommendations for BTT are proposed.

## 1. Introduction

Screening for *β*-thalassemia trait (BTT) depends on measuring hemoglobin (Hb) A_2_ accurately. However, since many factors like iron deficiency, *α*-thalassemia, *β*-gene mutations, gender, and smoking may affect HbA_2_ levels, the screening of BTT can be compromised [1–5]. The United Arab Emirates (UAE) is a multiethnic country with a BTT screening program because of a heavy burden of *β*-thalassemia disease. Consanguinity is common and marriages in the native population are arranged within the same tribe, which restricts gene flow and produces a heterogeneous distribution of BTT [6, 7]. As a preventive measure, mandatory federal premarital screening has been instituted throughout the UAE. In this population, though iron deficiency is common, iron stores are not routinely evaluated during the screening for BTT. In addition, *α*-thalassemia mutations are also frequent; this is important for BTT screening since their coinheritance with *β*-thalassemia mutations may lower the level of HbA_2_. Furthermore, *α*-thalassemia alters MCV and MCH, which adds to the risk of a missed diagnosis of BTT [2, 8]. In the UAE, 44 different *β*-thalassemia mutations have been reported though their phenotype, and the prevalence of silent mutations have not been systematically investigated [9–13]. Moreover, in this population the differences in lifestyle between genders might affect HbA_2_; for example, smoking is commoner among men, and the effect of smoking on HbA_2_ has not been studied. Women in this society are more prone to chronic nutritional disorders (e.g., obesity and type 2 diabetes), which may cause a high prevalence of iron deficiency [14]. The aim of this study was to investigate some of potential factors affecting HbA_2_ and its implications for BTT screening.

## 2. Methods

### 2.1. Setting and Study Population

The subjects (*n* = 1356) of this study were healthy young UAE adults, who were planning to marry; as mentioned earlier, premarital screening for BTT is mandatory and government funded. Data were collected between 2007 and 2012 in the screening center in the city of Al Ain, Abu Dhabi, UAE. During two six-month long window periods, data on 965 and 391 consecutive subjects, respectively, were collected. The characteristics of some of the study subjects were previously reported [15]. The study was approved by Al Ain Medical District Human Research Ethics Committee, and all participants signed a written consent.

### 2.2. Laboratory Tests

Complete blood count was performed on blood samples collected in EDTA tubes and was analyzed on the Cell-Dyn Sapphire (Abbott Diagnostics, USA) analyzer. Hemoglobin fractions were measured using high-performance liquid chromatography (Variant II, Biorad Co.) and capillary method (Sebia Co., France); the results of the two methods are comparable [16]. All screening tests were performed in the same laboratory at Al Ain Hospital, Al Ain, Abu Dhabi; the hospital subscribes to the United Kingdom National External Quality Assessment Scheme. All analytes used in the study met internal and external quality standards. Serum ferritin levels were determined in 391 consecutive individuals by an electrochemiluminescence technique using the Roche e411 Cobas immunoassay analyzer (Roche Diagnostics, Mannheim, Germany) in the research laboratory of the College of Medicine and Health Sciences, UAE University, Al Ain, Abu Dhabi, UAE.

### 2.3. Diagnostic Criteria

DNA gene tests were not performed in the study. BTT was documented when subject had the HbA_2_ > 3.5%, MCV < 80, and there was no abnormal hemoglobin variant present. Red cell microcytosis was defined as mean corpuscular volume (MCV) <80 fL. Two definitions for iron deficiency were used: (1) ferritin < 15 *μ*g/L (which is more specific) and (2) ferritin < 30 *μ*g/L (which is more sensitive) [17]. *α*-thalassemia trait was phenotypically defined when a subject had (1) MCV < 80 fL, (2) HbA_2_ ≤ 3.5%, (3) ferritin ≥ 30 *μ*g/L, and (4) RDW ≤ 14.0. Subjects with MCV > 80 fL, HbA_2_ ≤ 3.5%, ferritin ≥ 30 *μ*g/L, and RDW ≤ 14.0 were considered to have a normal genotype. In this population, *α*-thalassemia trait is due to *α* ^+^-deletions and mutations [8, 18]. Kinship groups (tribes) were defined by family names of study subjects and were coded to preserve confidentiality. A trained nurse took the basic clinical history documenting the smoking status, gender, and a family history of thalassemia.

### 2.4. Statistics

Standard descriptive statistical methods were used. In analysis, cutoff value of >3.5% was used to separate normal from elevated HbA_2_ levels. Student-*t* test was used to compare means of HbA_2_ in both groups with other variables and between subgroups. Chi-squared test was used for no parametric variables. ANOVA test was used to test the equality of HbA_2_ means between major tribes. Statistical significance was defined with two-tailed *P* value less than or equal 0.05. Data were coded and analyzed with SPSS statistical software version 19.0 (Chicago, Il, USA).

## 3. Results

### 3.1. Sex, Age, and Subjects with and without BTT

**Figure 1:**
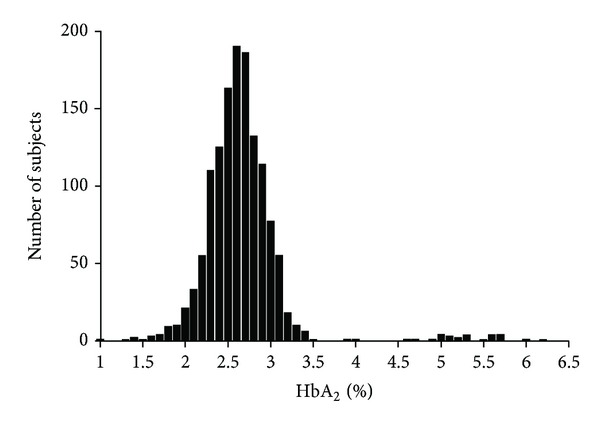
Distribution of HbA_2_ values in 1,356 subjects.

Of 1356 subjects, 50.6% were female. The mean age of males (26.0 ± 6.7 years, range 16–69) was three years higher than that of the females (22.9 ± 4.6 years, range 11–44). Among these 1356 consecutive cases, 29 (2.1%) had BTT. The distribution of HBA_2_ is shown in Figure 1.

### 3.2. Hemoglobin A_2_ in Non-BTT

In 1,327 subjects without BTT, the mean HbA_2_ level was 2.61 ± 0.31%.

#### 3.2.1. Hemoglobin A_2_ in Iron Deficiency

In all 77/379 (20.3%) iron deficient subjects, defined as ferritin <15 *μ*g/L, the mean HbA_2_ (2.30 ± 0.23%) was 0.2% lower than in subjects without iron deficiency (2.50 ± 0.24%, *P* < 0.0001). When iron deficiency was defined as ferritin <30 *μ*g/L, iron deficient individuals (138/379) had a mean HbA_2_ (2.39 ± 0.25%) that was 0.11% lower than in individuals without iron deficiency (2.50 ± 0.24%, *P* < 0.0001). In 75/198 (38%) iron deficient females (ferritin < 15 *μ*g/L), the mean HbA_2_ (2.31 ± 0.23%) was 0.17% lower than in iron sufficient females (2.48 ± 0.25%, *P* < 0.0001). When iron deficiency in females was defined as ferritin <30 *μ*g/L, the mean HbA_2_ (2.38 ± 0.25%) was 0.1% lower than in noniron deficient females (2.48 ± 0.26%, *P* = 0.01). Only 2 of 190 males had ferritin <15 *μ*g/L.

#### 3.2.2. Hemoglobin A_2_ in *α* ^+^-Thalassemia Trait

The phenotypically derived diagnosis of *α*-thalassemia trait is more reliable in males because they are rarely iron deficient. In 65 males with phenotypic *α* ^+^-thalassemia trait, the mean HbA_2_ (2.44 ± 0.20%) was 0.13% lower than in 107 without phenotypically derived *α* ^+^-thalassemia trait (2.57 ± 0.23%, *P* < 0.0001).

#### 3.2.3. Hemoglobin A_2_ in Men and Women

In the entire cohort without BTT, HbA_2_ levels for females (2.68 ± 0.65%) were lower than males (2.83 ± 0.71%, *P* < 0.0001). However, in subjects with ferritin >30 *μ*g/L, the mean HbA_2_ in females (2.52 ± 0.22%) was not different from males (2.57 ± 0.23%, *P* = 0.10) implying that sex does not affect HbA_2_.

#### 3.2.4. Hemoglobin A_2_ in Smokers

Males smoked tobacco considerably more often (39%, 68/174) than females (2%, 4/171). The red cell count, mean Hb, and mean HbA_2_ levels were not different between male smokers and nonsmokers (*P* = 0.42–0.76). 

#### 3.2.5. Hemoglobin A_2_ in Kinship Groups

The mean HbA_2_ level in subjects without BTT did not differ between 14 tribes comprising 62% of subjects (data not shown).

#### 3.2.6. Hemoglobin A_2_ in Borderline Range

**Figure 2:**
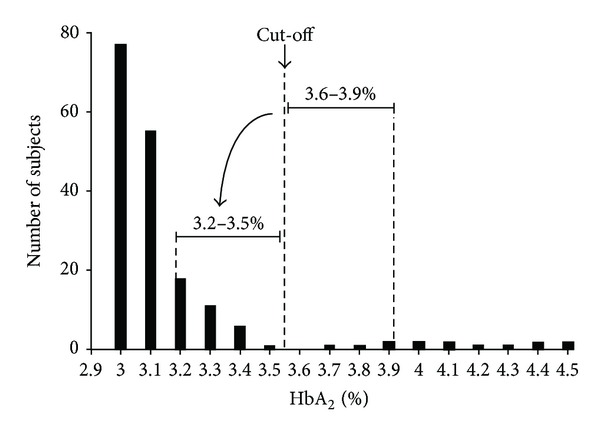
Iron deficiency may shift HbA_2_ value during screening resulting in misdiagnosis of BTT.

In iron deficient subjects (ferritin < 15 *μ*g/L), two standard deviations of HbA_2_ value are 0.4%. Thus, if individuals with BTT and HbA_2_ values between 3.6% and 3.9% are iron deficient, they could have nonthalassemic values of HbA_2_ that are between 3.2% and 3.5%. They are at a risk of false negative diagnosis of BTT (Figure 2). We found that 35 of 1356 subjects (2.6%) have HbA_2_ in the range of 3.2% and 3.5%, a group in which information about iron status is vital and most critical.

### 3.3. Hemoglobin A_2_ in BTT

In 29 subjects with BTT, the mean HbA_2_ was 5.2 ± 0.5%, with values ranging from 3.9% to 6.2%.

#### 3.3.1. Hemoglobin A_2_ in BTT and Iron Deficiency

Among 12 informative cases of BTT, five with iron deficiency (ferritin < 15 *μ*g/L) had lower mean HbA_2_ (5.24 ± 0.23%) than seven without iron deficiency (5.41 ± 0.45%, *P* = 0.45). 

## 4. Discussion

This study substantiates that HbA_2_ is lower if iron deficiency or the *α*-thalassemia trait are present; it is unaffected by gender, smoking, or tribal allegiance. However, many important questions remain: How valid are these findings? Can they be extrapolated to subjects with BTT? How should these observations impact screening for BTT? 

HbA_2_ was lower in iron deficient BTT carriers compared to noniron deficient BTT carriers; however, the difference was not statistically significant. This finding can be attributed to the small sample size or heterogeneity of *β*-thalassemia mutations. In subjects without BTT, HbA_2_ was lower in individuals who were more iron depleted (ferritin < 15 *μ*g/L) than subjects who were less iron depleted (ferritin < 30 *μ*g/L). This suggests a “dose effect” of body iron stores on the level of HbA_2_. In this study, two standard deviation dispersions of HbA_2_ value around the mean were 0.4% for all subjects and 0.46% for females with iron deficiency. Therefore, subjects with iron deficiency and HbA_2_ between 3.2% and 3.5% could theoretically have BTT, that is, a false negative test. Of the screened population, 2.6% were within this range of borderline HbA_2_ values. As it is unknown how many of them have *β*-thalassemia mutation (false negative cases are presumed to be rare), the value of routinely evaluating iron stores during BTT screening cannot be ignored. Studies have shown that iron deficiency lowers (while iron repletion increases) HbA_2_; thereby, the diagnosis from non-BTT carrier may change to BTT carrier and vice versa (reviewed in 1). Since 38% of females and <1% of males in our population had ferritin <15 *μ*g/L, evaluating routinely iron stores (i.e., serum ferritin) among females alone seems appropriate to facilitate effective screening. In addition, serum ferritin (which is not routinely measured during screening) is often requested if borderline HbA_2_ is encountered. In our study population, additional reasons warrant evaluation of iron stores during premarital screening. A diagnosis (and subsequent treatment) of iron deficiency in our population would benefit a large number of females and subsequently their infants since most women become pregnant after marriage. Moreover, with the high prevalence of *α* ^+^-thalassemia trait, iron deficiency in females, and BTT in our population, knowledge about iron stores will help community physicians to differentiate between these disorders, all of which are common causes of microcytosis and anemia in our population [7, 15]. Thus, the additional cost of serum ferritin (added to premarital screening of females) in our population is justified due to the high number of women and their children who would benefit from it.

The high frequency of *α* ^+^-thalassemia in this population, one of the highest in the world, may also affect the diagnosis of BTT [15, 18]. We confirmed that subjects with *α*-thalssemia trait (phenotypically derived) have lower levels of HbA_2_, an observation also observed by others [2]. However, whether coinheritance of *α* ^+^-thalssemia affects HbA_2_ level in subjects with BTT is less certain. In a Chinese population with BTT, the HbA_2_ level was unaffected by coinheritance of one *α*-globin gene deletion and two *α*-globin gene deleted in *cis* (*α* ^0^) [1]. In our population, *α*-thalassemia trait is due to two *α*-globin gene deleted in *trans* (*α* ^+^) [8, 18]. In patients with BTT and coinherited *α*-thalassemia resulting in hemoglobin H disease, HbA_2_ was found to be normal or elevated [19]. A coinheritance of *β*-thalassemia and *α*-thalassemia may result in normal MCV and MCH, a type of silent BTT that could result in a false negative diagnosis of BTT [20]. In our study, only one of 91 subjects with BTT had normal MCV (data not shown). The prevalence of silent *β*-thalassemia mutations that result in normal level of HbA_2_ is unknown in our population, and such prevalence is presumed to be low. Our study was not designed to determine the frequency of such mutations. However, in some recent studies, the frequency of silent *β*-thalassemia mutations (resulting in normal HbA_2_ level) is reportedly as high as 17% in some populations with endemic BTT [21–23]. Moreover, high prevalence of both iron deficiency (38% in females in our study) and *α*-gene deletion carriers (49% in other study) in the same population increases the likelihood of their coexistence in BTT carriers [15, 18]. The combined effect of iron deficiency and *α* ^+^-thalassemia on HbA_2_ in BTT is unknown. Finally, recently it was found that tribalism, resulting in marriages within Kinship groups and heterogeneous distribution of BTT carriers in our population, dramatically increases odds of a BTT carrier marrying another BTT carrier [7]. Therefore, in premarital screening of our population, a combination of several factors increases the uncertainty of a false negative diagnosis of BTT. This justifies a DNA test for *β*-thalassemia mutation in all individuals with normal HbA_2_, who plan to marry BTT carriers.

In this study, we also noted that HbA_2_ levels were not statistically different between men and women without BTT after excluding iron deficient females from the analysis, finding which has been observed earlier [5]. In a study of BTT carriers, the mean value of HbA_2_ was also not different between males and females after the effect of iron status and type of *β*-thalassemia mutation were taken into account [1]. In general, there is no good physiological rational for sex-based differences of HbA_2_. Similarly, smoking in our study did not affect the level of HbA_2_, a finding which was also observed in one other study [5]. 

The type of *β*-thalassemia mutation is a predictor of HbA_2_ level [3, 4]. In one study, mutation type had a stronger influence on HbA_2_ level than iron deficiency [1]. Our study population has a high number of different *β* ^+^- and *β* ^0^-thalassemia mutations resulting in the heterogeneity of HbA_2_ levels (Figure 1) [10–13]. While normal HbA_2_ value did not differ between the tribes, our sample was small for this analysis in BTT carriers.

### 4.1. Limitations of the Study

In the study, we derived genotypes from phenotypes. Although we have previously shown that red cell parameters of phenotypically derived *α* ^+^-thalassemia trait are correlated with the parameters of *α* ^+^-thalassemia trait derived by genotyping, we could not diagnose coinheritance of *α*- and *β*-thalassemias from phenotypes [15]. Due to restricted funding, ferritin was not measured in the entire cohort; the number of BTT carriers with iron deficiency was small. In addition, we extrapolated findings from subjects without BTT to those with BTT, which may not be always appropriate. 

### 4.2. Conclusions

In this Arab population, the cumulative risk of all the factors that lower HbA_2_ (iron deficiency, *α* ^+^-thalassemia, and many uninvestigated *β*-thalassemia mutations) is higher than appreciated. Since both iron deficiency and *α* ^+^-thalassemia trait are common and both lower the level of HbA_2_, this decrease in HbA_2_ may cause some BTT carriers to be missed on screening. As iron deficiency is mostly confined to females, routinely measuring serum ferritin in women is justified. In addition, since marriage between two BTT carriers can be disastrous for the progeny, a DNA test for *β*-thalassemia mutation is warranted in all subjects without BTT who plan to marry BTT carriers. The benefit of this approach to BTT screening needs to be validated by larger studies.
